# Accelerometer measurement of upper extremity movement after stroke: a systematic review of clinical studies

**DOI:** 10.1186/1743-0003-11-144

**Published:** 2014-10-09

**Authors:** Marika Noorkõiv, Helen Rodgers, Christopher I Price

**Affiliations:** Stroke Research Group, Institute of Ageing and Health, Newcastle University, Newcastle upon Tyne, UK; Adeli International Rehabilitation Centre, Valge 13, Tallinn, 11415 Estonia

**Keywords:** Motion, Technology, Neurological conditions

## Abstract

The aim of this review was to identify and summarise publications, which have reported clinical applications of upper limb accelerometry for stroke within free-living environments and make recommendations for future studies. Data was searched from MEDLINE®, Scopus, IEEExplore and Compendex databases. The final search was 31^st^ October 2013. Any study was included which reported clinical assessments in parallel with accelerometry in a free-living hospital or home setting. Study quality is reflected by participant numbers, methodological approach, technical details of the equipment used, blinding of clinical measures, whether safety and compliance data was collected. First author screened articles for inclusion and inclusion of full text articles and data extraction was confirmed by the third author. Out of 1375 initial abstracts, 8 articles were included. All participants were stroke patients. Accelerometers were worn for either 24 hours or 3 days. Data were collected as summed acceleration counts over a specified time or as the duration of active/inactive periods. Activity in both arms was reported by all studies and the ratio of impaired to unimpaired arm activity was calculated in six studies. The correlation between clinical assessments and accelerometry was tested in five studies and significant correlations were found. The efficacy of a rehabilitation intervention was assessed using accelerometry by three studies: in two studies both accelerometry and clinical test scores detected a post-treatment difference but in one study accelerometry data did not change despite clinical test scores showing motor and functional improvements. Further research is needed to understand the additional value of accelerometry as a measure of upper limb use and function in a clinical context. A simple and easily interpretable accelerometry approach is required.

**Electronic supplementary material:**

The online version of this article (doi:10.1186/1743-0003-11-144) contains supplementary material, which is available to authorized users.

## Introduction

Body worn motion sensors provide an opportunity for non-invasive, objective and accurate observation of patients’ movements during research and clinical rehabilitation [1–6]. These range from simple physical activity monitors (e.g. pedometers) [1] to more complex instruments that can capture precise limb position in a three-dimensional space (e.g. a combined accelerometer-gyroscope-magnetic field) [2]. The continuous development of technology and data analysis methods has resulted in an increasing number of proof-of-concept and validation studies [3, 4]. However for motion sensor approaches to fully explore recovery mechanisms or assist clinicians during therapeutic decision-making, they must reflect patient activity in real world settings.

Being small, light and affordable, accelerometers are an attractive technology for measurement of upper extremity movement. As loss of arm function is common after stroke [5], they have already been used to record activity (i.e. the amount of use per time unit) [7] and/or discriminate active from inactive periods [6, 8]. Although the information derived from standard accelerometers is limited to changes in speed and direction, if therapeutic applications are demonstrated in hospital and home settings then a large number of patients could benefit.

A broad systematic review of accelerometer applications after stroke has previously reported that accelerometers yield valid and reliable data about the physical activity of patients, but the clinical implications were unclear because of the limited evidence available [9]. The search was undertaken in October 2008 and has not been repeated to include more recent publications which focus on clinically relevant outcomes. Subsequent studies have included a heterogeneous range of upper limb impairment and function measures, and used a variety of statistical approaches to examine for associations with different accelerometer data formats. Consequently it is difficult to appreciate the current state of development of clinical accelerometer applications.

The aim of this systematic review was to identify and summarise publications that have reported clinical applications of upper limb accelerometry amongst stroke populations within free-living hospital or home environments, and thereby make recommendations for future clinical practice and research. To maintain clinical relevance, we sought to include only studies from real-world settings which reported clinical assessments in parallel with motion data.

## Review

### Methods

We systematically searched the published literature regarding the use of upper limb accelerometry amongst stroke patients to answer the following questions:

1. What are the clinically relevant research applications of upper limb accelerometers in a free-living hospital or home environment?
2. What types of accelerometers have been described and how were they used?
3. How was data collected and analysed?
4. How do the data measurements obtained relate to clinical assessments in a way that would be useful for clinicians and researchers?

The inclusion criteria were peer-reviewed original studies that (1) used movement sensors in a stroke population from a free-living clinical or domiciliary setting, (2) created a kinematic description of upper limb movement from sensor data, (3) used stand-alone accelerometers which did not require an external reference point (e.g. an electromagnetic field), (4) compared the kinematic data with a clinical assessment of impairment or function. We did not include articles that reported data (1) only relating to validity, reliability or algorithm and/or sensor development without any clinical comparison, (2) from sensors used in a visual feedback system or video game, (3) reflecting modification of the accelerometer output by other devices (e.g. from a separate gyroscope), and (4) where movement was limited to a very specific environment, function and/or task e.g. during performance of a subset of upper limb motor tasks from the Fugl-Meyer Assessment [10] or only during reaching [7]. Due to the small number of publications anticipated, all research designs were included and study quality was not formally rated. However quality is reflected by the study information reported including participant numbers, population details provided, methodological approach taken, technical description of the equipment used, blinding of clinical measures and whether safety and compliance data were collected.

**Figure 1:**
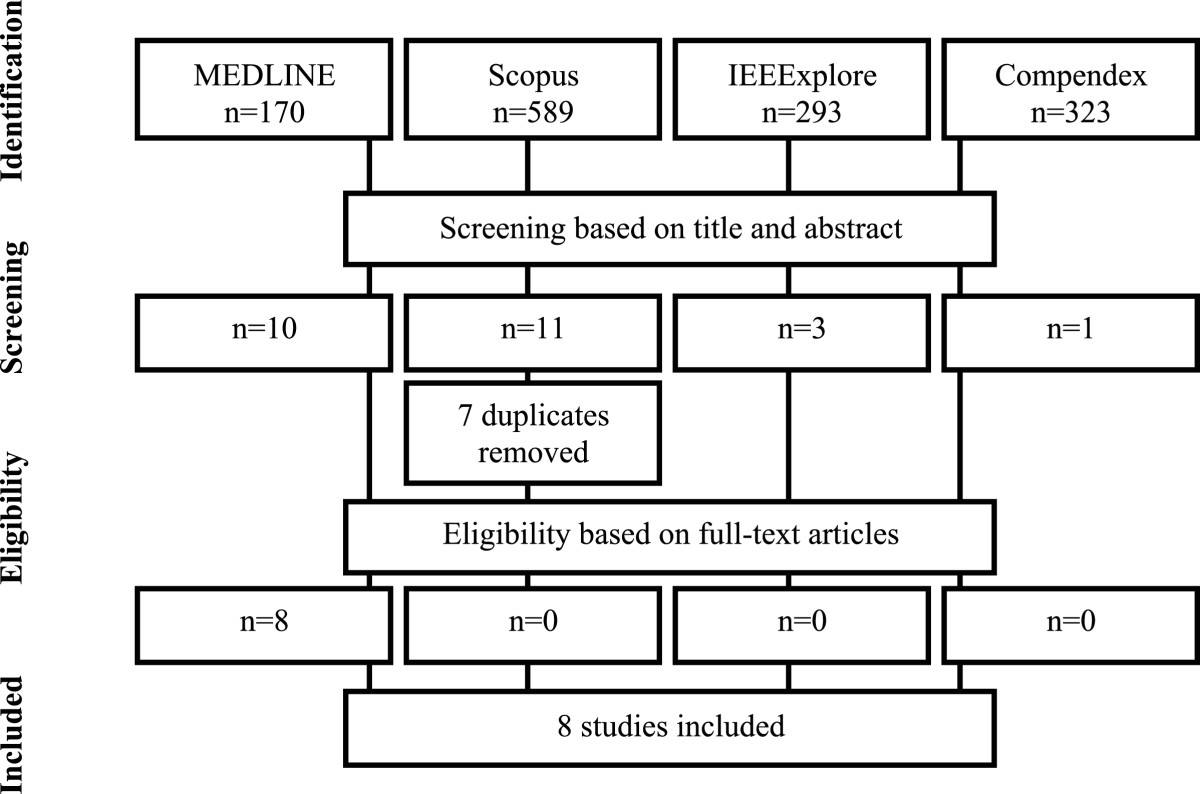
**Flow of information through the different search phases of a systematic review based on PRISMA 2009 guidelines.** Four search engines (i.e. MEDLINE, Scopus, IEEExplore and Compendex) were used to identify the relevant literature. After the screening based on title and abstract and removal of duplicates, 18 articles were selected. After the selection based on the full text article, 8 final articles were included in the current review.

The search strategy used for all databases is described in Appendix 1. Electronic databases were searched online through the Newcastle University library system: MEDLINE® (1946 to October Week 4 2013), Scopus (All years –October 2013), IEEExplore (1999–2013), and Compendex (1884–2014). The final search was 31st October 2013. The first author initially screened articles for inclusion based upon title and abstract, removed duplicates, retrieved full-text papers and selected final articles according to the inclusion and exclusion criteria (Figure 1). Inclusion of full text articles and data extraction was confirmed by the third author.

## Results

### Upper limb accelerometry applications in clinical stroke research

Eight articles fitted the inclusion criteria (Table 1). All included stroke patients without other neurological conditions represented. Three described rehabilitation interventions where accelerometer data were collected to explore mechanisms and/or as an outcome measure, including two randomised controlled trials. Age range was reported by three studies (35 to 94 years) [8, 11, 12] and the average age was reported by five studies [13–17] (Table 1). Gender distribution favoured male and was 65% overall. The inclusion criteria for studies were six mild to moderate [8, 11–14] and one severe [15] upper extremity impairment. The level of motor impairment was not reported [16]. Patients with excessive spasticity were excluded in two studies [13, 17], shoulder pain was an exclusion for two studies [15, 17] and wheelchair bound participants were excluded by one study [8]. Participants were required to have no or minimal cognitive deficit in all studies.

**Table 1:** **Overview of the selected articles**

	*Uswatte* et al. *(2006)*[6]	*Lang* et al. *(2007)*[11]	*Thrane* et al. *(2011)*[12]	*Van der Pas* et al. *(2011)*[8]	*Wang* et al. *(2011)*[13]	*Rand* et al. *(2012)*[16]	*Liao* et al. *(2012)*[17]	*Taub* et al. *(2013)*[15]
Aim	To study the reliability and validity of the Motor Activity Log for assessing real-world quality of a movement scale (QOM) and amount of use scale (AOU) of the hemiparetic arm in stroke survivors.	To determine the amount of arm use in people with hemiparesis post stroke during inpatient rehabilitation. To examine the relationships between upper extremity use, impairments and activity limitations.	To investigate the correlations between arm motor impairment and real-world use. To analyse whether arm movement ratio (AMR) is correlated with impairment or duration of arm use. To assess the influence of motor impairment on self-care activities.	To test whether triaxial arm accelerometry is a valid method to measure the amount of upper extremity activity in the daily life of adult stroke survivors.	To investigate the criterion-related validity, responsiveness, and clinically important differences of the ABILHAND questionnaire in patients with stroke.	To determine the change in daily use of the upper and lower extremities of stroke patients during subacute rehabilitation.	To compare the effects of robot-assisted therapy on real-world arm activity and daily function in a dose-matched (in amount of hours) control treatment group.	To determine whether patients with functionless hands would improve everyday use using a combination of Constraint-Induced Movement Therapy (CIMT) and conventional techniques for regulating tone.
Study design	Multicenter clinical trial.	Report.	Cross-sectional study.	Cross-sectional concurrent validity study.	Validation and clinimetric study.	Observational cohort.	Prospective randomized controlled trial.	Case series.
Blinding	Single-blinded (raters).	No.	No.	Not reported.	Blinded raters.	No.	Double-blinded	No.
Safety data	Not reported.	Not reported.	Not reported.	Not reported.	Not reported.	Not reported.	Not reported.	Not reported.
Sensors	Two-axial accelerometers (Manufacturing Technologies Inc., Fort Walton Beach, FL).	Uni-axial accelerometers (model 7164–2.4 Activity Monitors, MTI Health Services, FL).	Uni-axial ActiGraph GT1M Accelerometers (ActiGraph Inc., Pensacola, USA).	Tri-axial watch-like, water-resistant accelerometers (Actiwatch AW7a).	Accelerometers about the size of a large wristwatch. The type was not reported.	Tri-axial accelerometers (ActicalTM, Mini Mitter Co).	Tri-axial MicroMini-Motion logger (Ambulatory Monitoring, New York, NY, USA).	Accelerometers were not defined.
Placement	1 on each wrist.	1 on each wrist.	1 on each wrist.	1 around each wrist.	1 on each wrist.	1 on each wrist.	1 on each wrist.	1 on each arm.
Wearing time	3 days during all waking hours, except when in contact with water.	Single 24 h period, except for times when the devices would be exposed to water.	Single 24 h period.	Continuously for 3 days.	All day (not specified by authors for how long).	3 days on admission for rehabilitation and 3 weeks later prior to discharge. Could remove at night.	3 days before/after the intervention, except when in contact with large amounts of water.	3 days before and after each phase of the treatment.
Participants	222	34	31	45	51	60	20	6
Age	62.2 ± 13.0	63.9 ± 14.8 (range: 39–94)	65 ± 14	59.4 ± 9.2 (range: 39–80)	55.26 ± 10.31	61.0 ± 13.3	55.51 ± 11.17	56.9 ± 9.8
Men (%)	64	41	71	64	67	68	60	83
Time since stroke	3-12 months	9.3 ± 4.2 days	10.6 ± 6 days	2.6 ± 1.6 years	17.57 ± 13.43 months	33.4 ± 2.7 days	23.90 ± 13.39 months	5.1 ± 6.8 years
Setting	Outside the laboratory.	During in-patient rehabilitation.	Normal community-dwelling activity.	Normal community-dwelling activity.	Normal community-dwelling activity.	Normal community-dwelling activity.	Normal community-dwelling activity.	Normal community-dwelling daily activity.
Accelerometer activity measures	The ratio of duration of more- to less-impaired arm activity. Duration of less-impaired-arm activity as a% of the recording period (the number of epochs in the less-impaired arm data with above-threshold values divided by the total number of epochs).	Duration of impaired and unimpaired upper extremity usage during a 24 hr period.	(1) Duration of arm use, (2) The ratio of arm use duration between the more and less affected arm (AMR).	(1) The total sum of acceleration counts during waking hours divided by the number of waking hours. (2) Bilateral arm activity: the ratio of the sum of daytime accele-ration of the impaired arm to the unimpaired arm.	Ratio of affected to unaffected arm recordings.	The mean activity counts for the upper extremity for 1) an entire day, 2) a PT session, 3) an OT session and 4) daily use not including the OT/PT sessions.	Ratio of mean activity between the impaired and unimpaired arm.	The ratio of more-affected to less-affected arm recordings.

Applications of accelerometers in the selected studies were grouped into four main categories: (1) description of upper limb usage after stroke [11]; (2) assessment of the effect of therapeutic interventions such as robot-assisted therapy [17], constraint-induced movement therapy [16] and sub-acute stroke rehabilitation [15]; (3) comparison of real-world upper limb use with various measures of upper limb impairment and function; and (4) examination of the reliability and validity of a specific clinical scale such as the ABILHAND questionnaire [13] and Motor Activity Log [14]. No studies were identified which used accelerometer output to modify a therapeutic intervention in the same cohort. Compliance with accelerometry was reported only in the study by Liao et al. [17]: “all 20 study participants completed the study and no discomfort when wearing the device was reported and overall compliance was good”.

### Accelerometer type, site and duration

Three studies used tri-axial accelerometers [8, 16, 17], one study two-axial [14], two studies uni-axial accelerometers [11, 12] and the type of the accelerometer were not reported by two studies [13, 15]. All participants wore accelerometers on both wrists for either a single 24 hr period [11–13] or 3 consecutive days [8, 14–17]. Only Lang et al. explained their choice of data collection period: “the 24 hour period was chosen because: 1) that is the length of the day, enabling to capture activity that might occur outside of the typical workday or therapy hours, and 2) people with hemiparesis and controls were less interested in participating or less-compliant with wearing them if they had to be worn for longer periods” [11].

### Accelerometry data collection and analysis

Data were collected either as the summed acceleration counts over a specified time [8, 14, 16, 17], or as dichotomized data representing the duration of active and inactive periods [11, 12]. Different approaches were reported for defining a unit of arm activity, with data capture epochs varying from 1 second to 1 minute. Activity of both impaired and unimpaired upper extremities were measured and reported in all studies. A ratio of impaired to unimpaired arm activity was calculated for all studies except Rand et al. [16] and Lang et al. [12]. Two studies explained their choice for using a ratio as a main outcome measure: Liao et al. [17] chose arm activity ratio because the mean activity value of each arm would be subject to interference by other factors, such as walking pattern [18]; Uswatte et al. [14] explained that accelerometers were worn on both wrists during a trial of constraint induced movement therapy because a single unit on the impaired arm might cue use of that extremity and thereby confound measurement of treatment outcome and previous validation studies had suggested that the ratio of recordings controlled better for variations in overall levels of physical activity [1, 19].

In two studies the participants were asked to keep a diary to assist with accelerometry data analysis [8, 12]: Thrane et al. [12] asked participants to note in a diary when accelerometers were removed, travel and sleep in order to exclude these periods from analysis. Van der Pas et al. [8] asked participants to record times for going to sleep/getting up and trigger an event marker on the device. In the study by Rand et al. [16], a participant completed diary was not used but the activity counts of arm swing while walking were eliminated (5 consecutive steps or more in one epoch) to increase data specificity for goal directed hand usage.

### Clinical measures vs. accelerometry data

The correlation between various clinical test scores and upper limb accelerometry was examined in five studies (Table 2). Accelerometry data correlated significantly with most clinical tests of impairment such as active range of motion [11], muscle strength [11], Fugl-Meyer Assessment subscale for upper extremity [11], and with clinical tests of upper extremity function such as Action Research Arm Test (ARAT) [11], Wolf Motor Function Test (WMFT) [11], Functional Independence Measure (FIM) for motor and upper extremity function [11], different scales of Motor Activity Log (MAL) [8, 14], Stroke Impact Scale (SIS) Hand Function Subscale [8], a test of manual ability of the upper extremity ABILHAND [13] and Nottingham Extended Activities of Daily Living (NEADL) [13]. A lack of correlation was found with the Modified Ashworth Scale [11], composite light touch [11], joint position sense [11] and SIS Mobility Subscale [8].

**Table 2:** **Comparisons of accelerometer data and clinical scales**

	*Uswatte* et al. *(2006)*[16]	*Lang* et al. *(2007)*[10]	*Thrane* et al. *(2011)*[12]	*Van der Pas* et al. *(2011)*[8]	*Wang* et al. *(2011)*[13]	*Rand* et al. *(2012)*[16]	*Liao* et al. *(2012)*[17]	*Taub* et al. *(2013)*[15]
Statistical approach	Type 3,1 intraclass correlations.	Spearman correlation	Spearman correlation	Spearman correlation	Pearson correlations	Paired *t*-test	ANCOVA	Paired *t*-test
Accelerometer data comparison	AMR	Duration of use	Duration of use Calculated AMR	Activity counts Calculated AMR	Calculated AMR	Activity counts, the upper extremity activity did not change.	Calculated AMR. The robot-assisted therapy group improved compared with the active control group: accelerometer F_1,16_=5.91, p=0.026, effect size r=0.26.	Calculated AMR. Improved from baseline to post-intervention: t=2.9, p=0.016, d’=1.2
**Clinical Measures of Impairment**								
AROM		Shoulder flex r=0.30, p<0.05, elbow flex r=0.50, p=0.01, wrist ext r=0.63, p<0.01						Changed from baseline to post-intervention: t=6.1, p=0.001, d’=2.6.
FMS			The duration of use of the affected arm: r=0.60, p<0.001. AMR: r=-0.85, p<0.001			FMA improved: t=-2.9, p=0.005	The robot-assisted therapy group improved compared with the active control group: FMA F_1,16_=14.32, p=0.002, effect size r=0.46	Change in FMA from baseline to post-intervention t=4.0, p=0.005, d’=1.6
Modified Ashworth Scale		r=-0.31, n.s						
Pain		Shoulder pain r=0.41, p<0.01						
Sensation		Composite light touch r=-0.15, n.s., joint position sense r=-0.03, n.s						
Strength using a hand-held dynamometer		Shoulder flex r=0.34, p<0.01, elbow flex r=0.52, p<0.01, wrist ext r=0.37, p<0.01, grip r=0.42, p<0.01						
Gait speed						Improved: t=-4.8, p<0.001		
**Clinical Measures of Function**								
ARAT		r=0.40, p<0.01				Improved: t=-4.7, p<0.001		
FIM		Motor r=0.67, p<0.01, UE r=0.58. p<0.01.				Improved: t=-7.6, p<0.001	The robot-assisted therapy group improved compared with the control group: FIM F_1,16_=0.03, p=0.88, effect size r=0.002	
WMFT		Function r=0.62, p<0.01; time r=-0.65, p<0.01						
BBS						BBS improved: t=-6.4, p<0.001		
6MWT						6MWT improved: t=-4.8, p<0.001		
**Clinical Questionnaires**								
ABILHAND					At baseline and post treatment: r=0.45–0.54, p<0.01.		The robot-assisted therapy group improved compared with the control group: F_1,16_=4.76, p=0.043, effect size r=0.22	
MAL	AMR was correlated with QOM r=0.52, p<0.01 and AOU r=0.47, p<0.01. Less-impaired arm accelerometry was not correlated with QOM r=0.14, n.s. and AOU r=0.14, n.s.			Bilateral arm activity (mean of 2 arms): MAL-26AOU Scale r=0.37, p<0.01. MAL-26AOU Scale r=0.37, p<0.01.			The robot-assisted therapy group improved compared with the control group: MAL AOU F_1,16_=9.39, p=0.007, effect size r=0.36, MAL QOM F_1,16_=13.48, p=0.002, effect size r=0.44	Change in FL-MAL Arm Use scale from baseline to post-intervention: t=7.4, p=0.001, effect size(d’)=3.0
				AMR: MAL-26AOU Scale r=0.60, p<0.001, MAL-26QOM Scale r=0.66, p<0.001.				
				Affected arm activity: MAL-26AOU Scale r=0.58, p<0.001, MAL-26QOM Scale r=0.65, p<0.001.				

Note: AMR – arm movement ratio, AROM - Active Range of Motion, ARAT – Action Research Arm Test, WMFT – Wolf Motor Function Test, FIM – Functional Independence Measure, FIM UE – FIM Upper Extremity, FMS – Fugl-Meyer Scale, MAL – Motor Activity Log, LF-MAL – lower functioning MAL, MAL-26 QOM – MAL-26 Quality of Movement, MAL-26 AOU – MAL-26 Amount of Use, CIMT – Constraint-Induced Movement Therapy, SIS – Stroke Impact Scale, NEADL – Nottingham Extended Activities of Daily Living, BBS – Berg Balance Scale, 6MWT – 6 Minute Walking Test.

Consistent with studies in controlled settings, Thrane et al. [12] found a stronger correlation between a clinical assessment of impairment (Fugl-Meyer Assessment (FMA) and the movement duration ratio between the least affected and more affected arm (r = -0.851, p < 0.001) than between the FMA and raw accelerometer data (r = 0.601, p < 0.001). However Van der Pas et al. [8] found a high correlation between unilateral (affected arm) and bilateral (ratio) accelerometry (the number of counts per time unit representing movement ‘intensity’) and MAL-AOU (amount of use) and MAL-QOM (quality of movement) scales, suggesting that both unilateral and bilateral arm accelerometry are valid methods for assessing arm activity related to function in daily life of patients after stroke.

Thrane et al. [12] found a variation in arm movement ratio (AMR) from accelerometer readings especially in the upper range (less impairment) of FMA: in the FMA range of 43–47, the AMR varied from a normal level of 1.2 to 2.5, indicating 2.5 times greater movement of the less-affected arm which could indicate perceptual difficulties or “learned non-use” amongst some participants. No cofactors interfered with this relationship in the two-factor regression models (age, gender, days since stroke, initial stroke severity, apraxia, and lower extremity function). Hand dominance and choice of tasks could also explain AMR variations in subjects with the same level of motor function, however incomplete data prevented the authors from further exploration of this finding and the clinical implication is not clear [12].

The efficacy of an intervention was tested in three studies [15–17] where accelerometry and clinical tests data were collected in parallel. In two of these, the accelerometer data ratio between the impaired and unimpaired arm and clinical test scores both detected a difference from baseline to post-treatment [15, 17]. The FMA [15, 17], MAL [15, 17], Functional Independence Measure [17] and active range of motion of the upper limb [15] were used as outcome measures. However, in the study by Rand et al. [16], there was a discrepancy between the clinical test scores and accelerometry: clinical test scores showed motor and functional improvements while upper extremity accelerometry data (mean activity counts) did not change (Table 2). Although this discrepancy may reflect methodological factors, accelerometer data might be a better representation of real world activity, which is influenced by many more factors than just motor performance.

## Discussion

Measurement of upper extremity movement by accelerometry during day-to-day activities in hospital or at home has the potential to provide additional valuable information about recovery after stroke. The eight studies identified for the present review, including a large multicentre clinical trial (n = 222) [14] and a prospective randomised controlled trial (n = 20) [17], have demonstrated that accelerometry can be systematically applied as a method for measuring overall upper extremity activity. However it is still uncommon that motion sensors are used to inform the development of new clinical rehabilitation approaches.

Accelerometry data correlated well with the clinical tests of impairment and function, apart from measures which do not directly reflect upper limb motor function such as tone, sensation and mobility. Only the study by Rand et al. [16] reported that accelerometry failed to detect change in upper extremity usage after a rehabilitation programme whilst clinical tests showed a significant decrease in impairment and improved function. Interestingly, this was one of the two studies where the ratio of impaired to unimpaired arm usage was not calculated and reported the usage of impaired upper extremity only, but it should be also considered that rehabilitation approach, patient motivation and learned non-use might be reasons why clinical recovery is not reflected in daily upper limb activity [16]. Improvements in stand-alone clinical assessments without matching accelerometer readings might indicate that participants have not translated motor recovery into daily life. Rand et al. [16] suggested that the gap between the recovery of capacity (i.e. clinical measurements) and the lack of improvement in performance (i.e. daily use of the upper extremities according to accelerometer data) provides a useful guide for clinicians. If this is demonstrated by appropriately designed studies then feedback of accelerometer data to patients and clinicians may have a therapeutic role. Improving the interpretation of accelerometry data with clinical questionnaires (e.g. the Motor Activity Log and ABILHAND) could allow researchers to capture a wider spectrum of change in daily function for stroke patients receiving rehabilitative therapies [8, 13, 14, 17]. In several studies participants were asked to keep a diary describing their everyday activities such as periods of walking, sitting, driving car etc. [8, 12]. However, using activity log sheets in addition to accelerometry will set certain limitations for study recruitment due to the extra time, effort and motivation required from participants and researchers, and is only feasible if the participant can write, or a carer can reliably complete log sheets on their behalf.

Accelerometry data were collected either during 24 hours or 3 consecutive days, which was justified by only one author [11]. Other studies mostly referred to the published protocols by Uswatte et al. [1, 6] where data collection is over three consecutive days, possibly because the accelerometer continuous recording capacity is approximately 72 h. It is unclear how long accelerometry data should be collected for in order to obtain a meaningful description of upper extremity usage in a real-world environment [20, 21]. It has been suggested that 7 to 10 and 14 to 21 days of assessment are required to reliable observe (i.e. R = 0.80), total activity in healthy men and women, respectively [22]. In contrast, 21 to 28 days are required for reliable observation of non-occupational activity patterns in healthy men and women [22]. Measuring physical activity levels and patterns of patients during neurological rehabilitation will be more challenging, as the duration and amplitude of activities will be limited by changes in motor function [11]. In community settings it has been shown that adults participants are more active on Saturdays than Sundays [22, 23], and for a real-world examination of motor behaviour it would be important to balance the days of the week [21].

Further interpretation of data still has limitations, mainly because of contamination by other activities that did not include purposeful arm movement e.g. walking. Attempts have been made to overcome these limitations by using special data collection and analysis methods to prevent overestimation of upper extremity use. Most of the studies included in the present review defined upper extremity use as any time when accelerometer data suggested movement. However there were alternative approaches. Rand et al. [16] defined upper extremity usage as the number of activity counts during a specified time. Liao et al. [17] defined upper extremity usage as the area under the transducer signal curve proposing that this detected subtle change in hemiplegic arm activity and so was more appropriate for rehabilitation studies than a threshold approach [9, 24].

Six studies out of eight used the ratio of impaired to unimpaired arm as the outcome of upper extremity usage [8, 11–14, 17], three of the six studies reported the ratio only [13, 15, 17]. Whilst the ratio can aid correction for the variability in overall physical activity, its interpretation is limited if the absolute activity of impaired and unimpaired arms is not reported. For example, Lang et al. [11] found that the activity duration of the impaired arm was less than unimpaired, however, the usage of both was less than that of healthy controls. They also found that the hours of dominant and non-dominant upper extremity use were not different [11] and that affected and unaffected upper extremity use was positively correlated, supporting observations that upper extremities are most often used together to perform bimanual tasks during daily activity [11]. In addition, Van der Pas [8] found that movement of the affected arm after stroke was related to performance of functional activities when that arm was dominant pre-stroke. Therefore when upper extremity usage is reported as a ratio, the underlying data is still dependent upon intra- and inter-individual variations in bilateral upper extremity activity. The personal choice of tasks attempted will also influence conclusions which can be drawn when comparing between individuals in real-world settings, which has implications for use of accelerometry during rehabilitation programmes where patient and therapist select repetitive movements from a menu, e.g. [25].

The type of sensors varied from uni- to multiple-axis accelerometers. Although a strong positive correlation between the output from uniaxial and multiple axis accelerometers have been reported [2], the validity coefficients reported for multiple axis units have been higher than those reported for uniaxial models [2]. Importantly, it is impossible to calculate a minimal clinical difference for arm use by a uniaxial accelerometer because they only measure in a single plane of movement [9]. Thus, multiple axis accelerometers should be preferred to uniaxial devices.

### Study limitations

Although we sought to identify studies in free-living rather than controlled laboratory settings, the patients included were still not typical of the stroke population, being younger than the average age of stroke occurrence in UK (~ 75 y) [26] and more often male. There was a tendency to use accelerometry amongst patients with moderate to mild impairment. Time since stroke varied across a large range and it is likely that contact with rehabilitation services would have varied similarly. There was no data safety or tolerance reported (except the comment by Liao et al. [17]). These factors reduce the relevance of current evidence for typical neurological rehabilitation populations. Identifying and overcoming difficulties that limit the use of real-world accelerometry across a whole range of patients will be a challenging but an important focus for future studies.

## Conclusions

Real-world usage of the upper extremity during stroke rehabilitation is still not yet well described and we require better knowledge of how to interpret different variables of accelerometry against clinical measures which holds meaning for clinicians and patients. Recommendations from this review of recent studies are:

- Clinical measures are still required to provide context for interpretation in case the individual’s recovery is not reflected through real world accelerometer data e.g. due to learned non-use
- The ratio between impaired and unimpaired sides is the standard approach for upper limb accelerometry but hand dominance might require further consideration depending upon individually chosen rehabilitation goals
- Diaries should be used for at least a proportion of the monitoring period in order to relate individual accelerometer data to background levels of activity.
- Simple, user-friendly cost-effective and easily interpretable upper limb accelerometry methods are still required if this is to be a useful tool to monitor patients’ progress alongside clinical assessments of motor recovery.

## Appendix 1: search strategy

MEDLINE: (stroke* and rehabilit* and accelerometer*).mp. [mp = title, abstract, original title, name of substance word, subject heading word, keyword heading word, protocol supplementary concept, rare disease supplementary concept, unique identifier]

Scopus: TITLE-ABS-KEY(stroke* AND rehabilit* AND accelerometer*)

IEEExplore: stroke* and rehabilit* and accelerometer*

Compendex: ((((stroke*) WN KY) AND ((rehabilit*) WN KY)) AND ((accelerometer*) WN KY))

## Authors’ original submitted files for images

Below are the links to the authors’ original submitted files for images. Authors’ original file for figure 1
