# Supplementary figures and images for: Accelerometer measurement of upper extremity movement after stroke: a systematic review of clinical studies
Source: J Neuroeng Rehabil. 2014 Oct 9;11:144. doi: 10.1186/1743-0003-11-144 (PMC4197318; doi:10.1186/1743-0003-11-144)

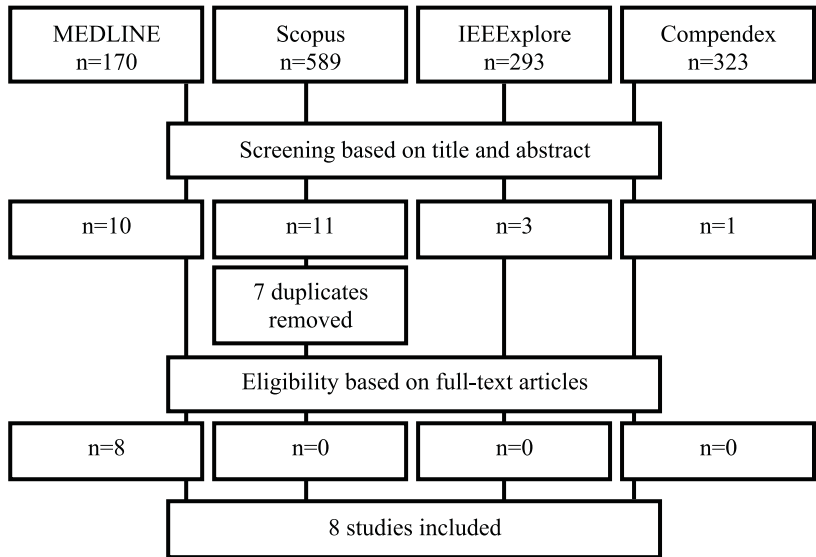

Supplement: Supplementary file 1 — Authors’ original file for figure 1 [file 12984_2014_663_MOESM1_ESM.pdf]
